# The Application of Anatomy Combined With Ultrasound Knife in Transaxillary Endoscopic Biplane Breast Augmentation

**DOI:** 10.3389/fsurg.2022.865379

**Published:** 2022-04-27

**Authors:** Jiachao Xiong, Qiang Hou, Zheyuan Hu, Yakun Gao, Lu Lu, Meiqing Sun, Hao Hu, Yuxin Qian, Hui Wang, Hua Jiang

**Affiliations:** ^1^Department of Plastic and Reconstructive Surgery, Shanghai East Hospital, School of Medicine, Tongji University, Shanghai, China; ^2^Department of Plastic and Reconstructive Surgery, Changzheng Hospital, Naval Medical University, Shanghai, China

**Keywords:** transaxillary, endoscope, biplane, breast augmentation, breast anatomy

## Abstract

**Objective:**

We aim to clarify the vascular and nerve anatomy of the breast and combine it with an ultrasound knife to use in transaxillary endoscopic biplane breast augmentation.

**Methods:**

This study is a retrospective review of patients undergoing transaxillary endoscopic biplane breast augmentation between October and October 2021. Related variables were collected using a standardized data collection template. The detailed process of the transaxillary endoscopic biplane breast augmentation under anatomy instruction is carefully described in this study, and the postoperative effect was closely observed.

**Results:**

Sixty-three female patients underwent transaxillary endoscopic biplane breast augmentation. The average implants volume counted 242.46 ± 31.34 cc, and the average operation time was 155.92 ± 22.34 min. Patients were followed up for a mean of 13.67 months (range, 3–27 months), and most of the patients achieved good postoperative results and no severe complications and were satisfied with both appearance and function.

**Conclusions:**

The application of anatomy combined with an ultrasound knife in transaxillary endoscopic biplane breast augmentation is a promising way to achieve good breast shapes with high patient satisfaction and is worthy of clinical promotion and application.

## Introduction

Women generally suffer from breast sagging and volume reduction due to breast atrophy after breastfeeding and congenital breast dysplasia. Prosthesis breast augmentation can promote breast enlargement and plumpness of symmetric shape, which is currently one of the most commonly used methods (1). The common implant pockets of prosthetic breast augmentation are dual-plane, subglandular, subpectoral, and subfascial breast, and the postoperative results are different. Dual-plane mammoplasty allows the prosthesis to be placed in the two planes behind the breast and pectoralis major at the same time, which has many advantages of two single planes and is the most widely used. The incision of transaxillary mammoplasty is located in the secret area of the body and better preserves the sensitivity of the nipple, which has become a popular breast augmentation method and is especially suitable for Asian women (2–4). However, the transaxillary approach tests the surgeon's prediction of the anatomical scope, and the prosthesis is difficult to be implanted through the narrow axillary passage and obtain obvious symmetrical inframammary folds, which is more likely to cause complications such as hematoma and infection (5). With the intervention of endoscopes, transaxillary mammoplasty with the assistance of endoscopes makes the anatomy of the implanted cavities under the pectoralis major more accurate and greatly reduces the occurrence of the above complications (6).

In this article, we have clarified the vascular and nerve anatomy of the breast, aiming to guide the transaxillary endoscopic biplane breast augmentation through the instruction of the anatomy. At the same time, we found that the application of ultrasonic scalpel in transaxillary endoscopic biplane breast augmentation greatly reduces the incidence of intraoperative bleeding and postoperative hematoma.

## Patients and Methods

In this study, 63 female patients who were admitted to the department of plastic surgery of our teams were included. All patients in this study were diagnosed with small breasts and decided to undergo transaxillary breast augmentation with endoscopy between October 2019 and October 2021. The inclusion criteria were as follows: (1) aged ≥18 years; (2) without ptosis; (3) appealed to avoid scarring on the breast. The exclusion criteria were patients with significant chest wall irregularities. Descriptive analyzes were performed and the results were presented as mean ± SD. This study was approved by the hospital ethics committee and all patients provided their informed written consent. In addition, the patient's operation time and cost were also recorded, and a breast-Q (version 2.0) questionary was performed to evaluate the patients' satisfaction.

## Surgical Technique

### Marking of Preoperative Key Indicator Measurement

**Figure 1:**
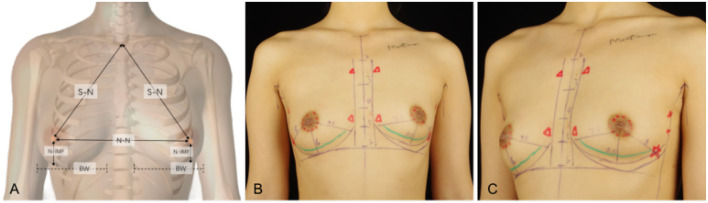
Some key indicators of the patients, including approximate projection of chest blood vessels and nerves on the body surface, inframammary sulcus line of the breast before and after breast augmentation, anterior axillary line, fovea jugularis, parasternal line, breast boundary line, distance from sternal notch to nipple (S-N), breast base width (BW), distance from the nipple to the inframammary fold (N-IMF), the distance between breasts (N-N). **(A)** Some indicators show in a schematic diagram. **(B)** Marking on the clinical case, a 26-year-old patient with a transaxillary breast augmentation with endoscopy. Breast augmentation with endoscopy under anatomy instruction frontal views. **(C)** Breast augmentation with endoscopy under anatomy instruction oblique views.

As shown in Figure 1, some key indicators of the patients, including approximate projection of chest blood vessels and nerves on the body surface, inframammary sulcus line of the breast before and after breast augmentation, anterior axillary line, fovea jugularis, parasternal line, breast boundary line, distance from sternal notch to nipple (S-N), breast base width (BW), distance from the nipple to the inframammary fold (N-IMF), the distance between breasts (N-N), soft tissue pinch test of the upper pole (STPTUP), and soft tissue pinch test of the inframammary fold (STPIMF), are critical to the selection of personalized breast prosthesis and determine the intraoperative dissection area and boundary. Therefore, patient standing position and the above indicators of the patient were measured and marked before the operation, and photographs were collected under the consent of the patient.

### Transaxillary Endoscopic Breast Augmentation

**Figure 2:**
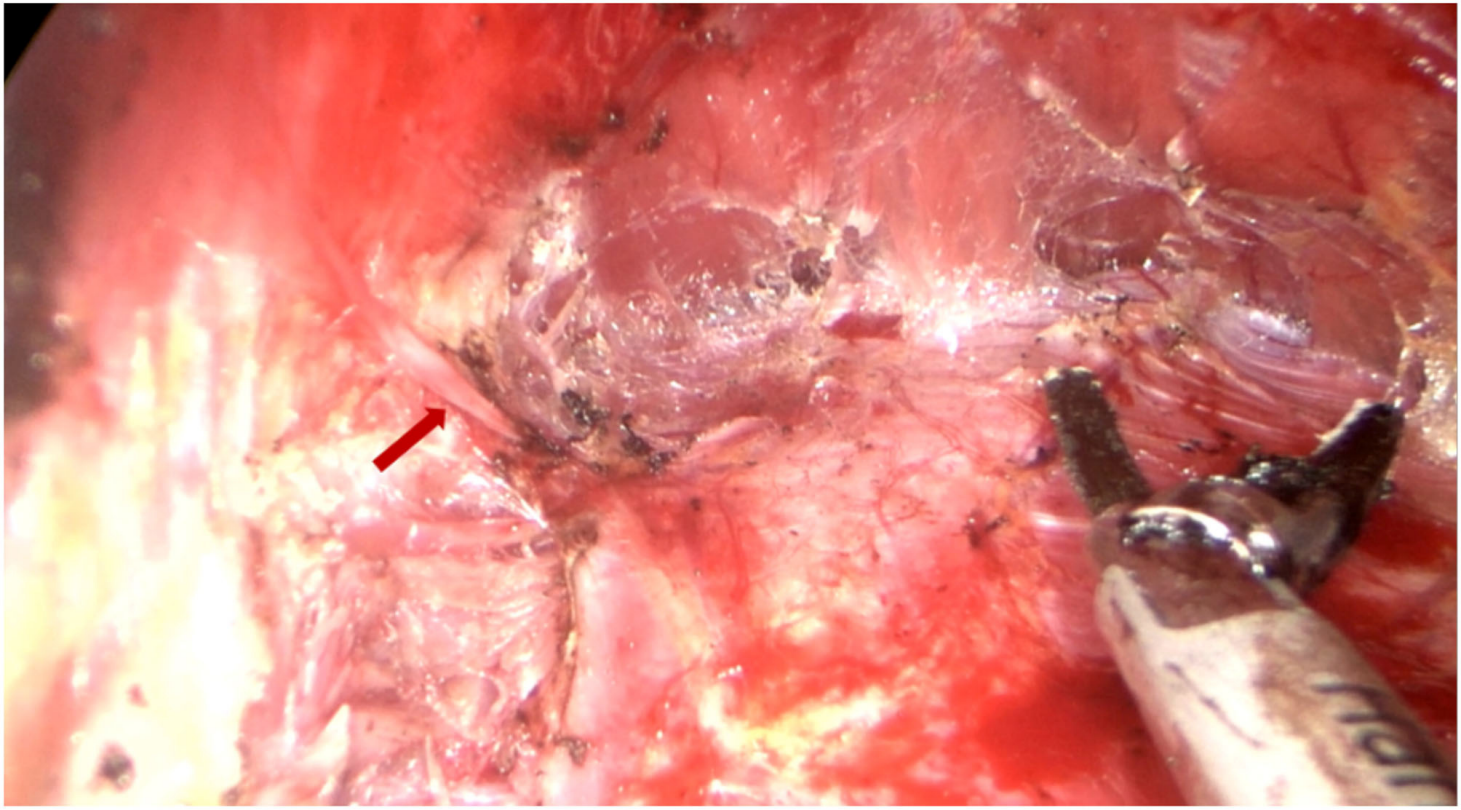
Dissecting the lateral pocket during endoscopic transaxillary breast augmentation. Near the anterior axillary line of the upper border of the fifth costal, the fourth intercostal anterior skin branch (indicated by the red arrow) can be clearly observed under the endoscope, which needs to be carefully retained.

**Figure 3:**
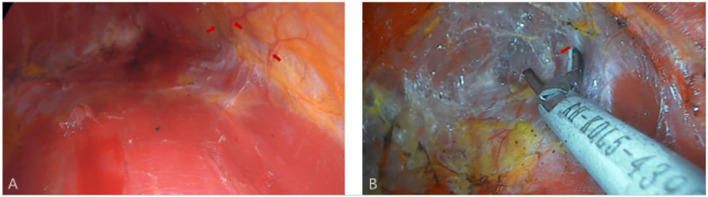
The medial and upper pockets were dissected during endoscopic transaxillary breast augmentation. **(A)** Arrows indicate multiple intercostal perforators of internal thoracic artery perforating vessels can be clearly observed in the second and fourth intercostal spaces. **(B)** Arrow indicates a deep blood vessel from the pectoralis minor to the pectoralis major near the second rib of the midclavicular line.

**Figure 4:**
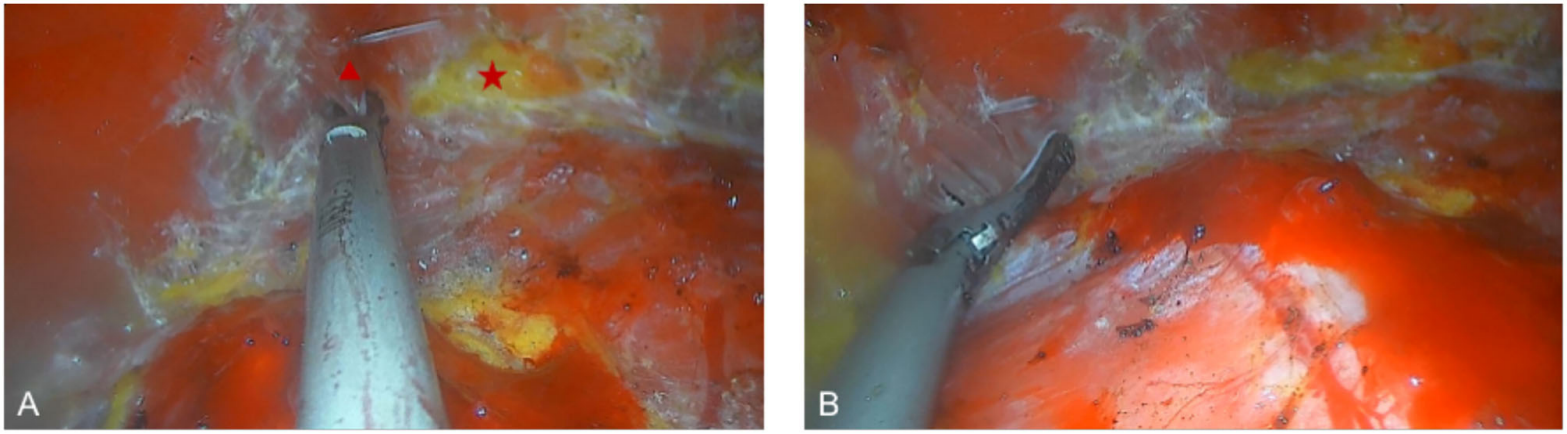
Dissecting the lower pocket during endoscopic transaxillary breast augmentation. **(A)** The attachment points of the pectoralis major around the inframammary fold were transected, the triangle and the star indicate the pectoralis major muscle, and the adipose tissue after the pectoralis major muscle is severed, respectively. **(B)** Transection of the pectoralis major muscle to separate the outer boundary of the pocket.

All surgical patients were supine with arms abduction at 90 degrees and received general anesthesia. The operation area was disinfected and covered with a sterile towel, nipple protective shields were performed, and marked the axillary incision line. A longitudinal skin incision line of approximately 3~4 cm was made in the axillary roof, and the subcutaneous tissue was cut and dissected to fully expose the fascia of the lateral border of the pectoralis major. The lateral fascia of the pectoralis major was incised and performed a blunt separation on the deep surface of the pectoralis major. Then, the endoscope retractor was inserted along the lateral posterior cephalic space of the pectoralis major. The endoscope was inserted and adjusted to clear the operation field. Under the clear vision of the endoscope, combined application of ultrasonic knife to construct the subpectoralis major cavity. For the lateral border of the cavity, the implanted lacuna was separated to the anterior axillary line to prevent the lateral displacement after implantation. Near the anterior axillary line of the upper border of the fifth costal, the fourth intercostal anterior skin branch can be clearly observed under the endoscope (Figure 2), which needs to be carefully protected to prevent damage to the nipple sensation. For the medial border, the implanted lacuna was dissected to the parasternal line and multiple intercostal perforators of internal thoracic artery perforating vessels can be clearly observed in the second and fourth intercostal spaces on the lateral edge of the sternum at. 5~1 cm away from the parasternal line (Figure 3A), and an ultrasonic scalpel was performed to fully cut them off. For upper bounds, there is a deep blood vessel from the pectoralis minor to the pectoralis major near the second rib of the midclavicular line, which needs to be fully cut them off (Figure 3B and Supplementary Video 1). For the lower bound, the implanted lacuna was separated to the new inframammary sulcus line of breast and the attachment points of the pectoralis major around the inframammary fold were transected, inside to the sternum, outside to the axillary front (Figure 4 and Supplementary Videos 2, 3). After the subpectoralis major cavity was constructed, the ultrasonic knife was used to fully stop the bleeding. Subsequently, the pectoralis major was transversely broken at about 1 cm from the insertion of ribs and internally to the parasternal line to form a biplane. Through the axillary incision, a conveyor belt was used to insert a prosthesis into the cavity, roll the operating table to 60 degrees to observe the shape and symmetry of the breast, and adjust until the breast augmentation result was satisfactory. A drainage tube was placed in the breast operation area and straticulateinterrupted sutures were applied to close the wound, and then wrapping with suitable pressure in the chest was used for shaping to prevent prosthesis displacement.

## Results

Patients underwent transaxillary retropectoral biplane breast augmentation with endoscopy, the average age of them was 18–58 years old, and shaped gel implants with an average size of 180–300 cc were used. The average operation time was 155.92 ± 22.34 min, and the average cost of surgery was 16,740.79 ± 373.96 RMB. And the cost of endoscopy and ultrasonic knife accounts for about 14% of the total cost. Detailed demographic information is shown in Table 1. The drainage tubes were removed when drainage decreased to <40 ml/24 h, all patients were discharged the next day after surgery and requested with restriction of shoulder exercise.

**Table 1:** Patient demographic characteristics.

**Characteristics**	**Quantity**
Age/year	31.30 ± 8.76
Height/cm	164.63 ± 4.68
Weight/kg	51.17 ± 5.78
BMI	18.87 ± 1.91
Implant brand	
Allergen	11
Allrua	2
Winner	1
Mentor	49
Implant volume/cc	242.46 ± 31.34
Operation time/min	155.92 ± 22.34
Operation cost/RMB	16,740.79 ± 373.96

**Figure 5:**
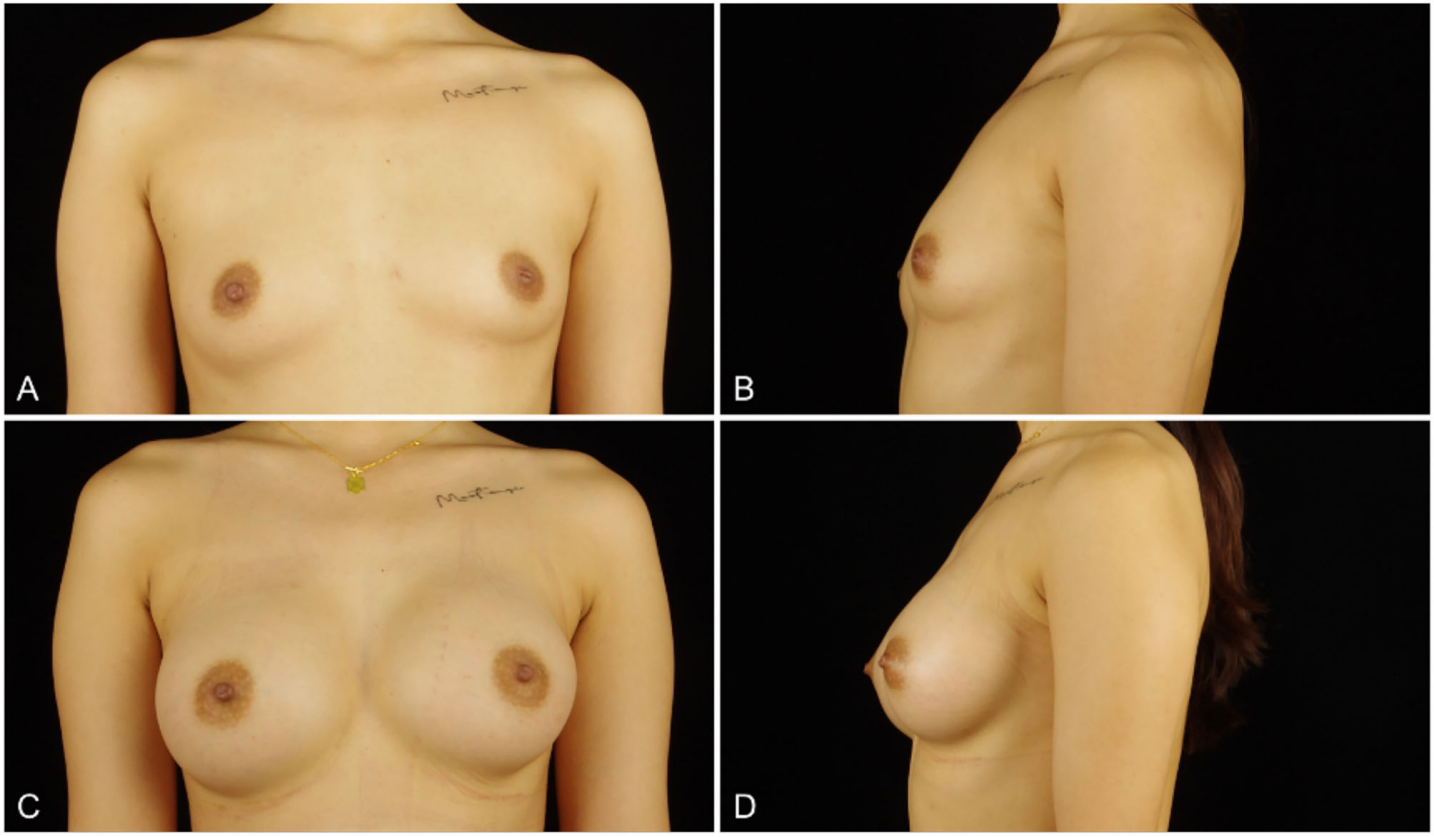
**(A,B)** a 26-years-old woman (height, 167 cm; weight, 55 kg; body mass index, 19.7 kg/m^2^) presented for endoscopic transaxillary breast augmentation with shaped gel implants (Mentor, smooth round moderate 295 cc implants, placed bilaterally). **(C,D)** Three months postoperatively.

**Figure 6:**
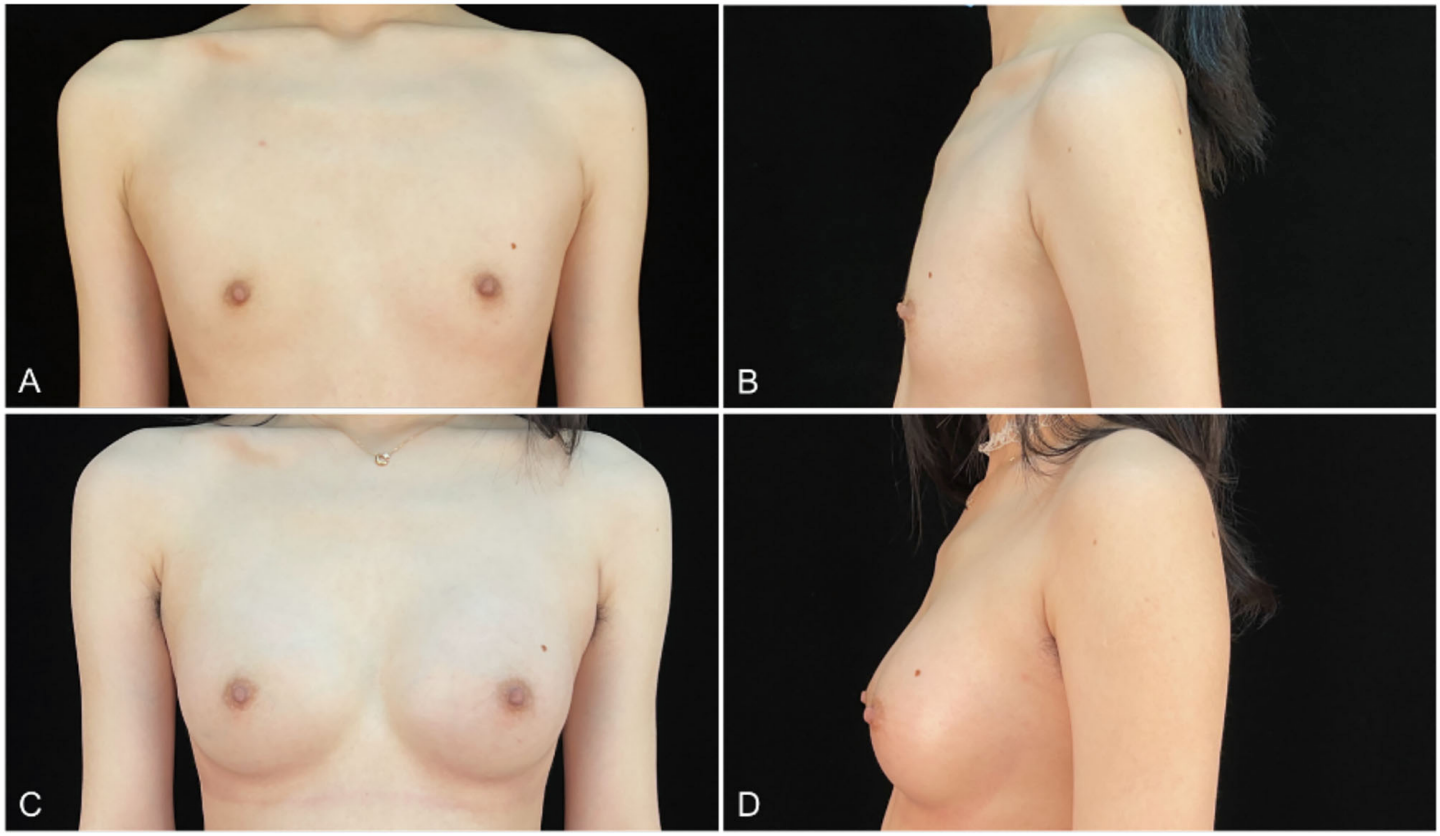
**(A,B)** an 18-years-old woman (height, 175 cm; weight, 56 kg; body mass index, 18.3 kg/m2) presented for endoscopic transaxillary breast augmentation with shaped gel implants (Natrelle, smooth round moderate 255 cc implants, placed bilaterally). **(C,D)** Six months postoperatively.

Patients were followed up for a mean of 13.67 months (range, 3–27 months). All patients have no nipple sensation function reduction and major complications such as serious bleeding, infection, breast implant rupture, implant malpositions, or serious asymmetrically. Capsular contracture was found in only one patient half a year after surgery. Two patients complained of slight asymmetry in the early stage but improved with shaping wrapping. One patient had a unilateral hematoma in the second week postoperatively (Table 2). The postoperative satisfaction of patients was investigated, and the results showed that 97% of patients were satisfied with the effect of breast augmentation (Figures 5, 6).

**Table 2:** Transaxillary endoscopic biplane breast augmentation complications (*n*, %).

**Complications**	**No. of patients (%)**
Nipple sensation function reduction	0 (0)
Serious bleeding	0 (0)
Infection	0 (0)
Breast implant rupture	0 (0)
Implant malpositions	0 (0)
Serious asymmetricality	0 (0)
Slight asymmetry	2 (3.2)
Capsular contractures	1 (1.6)
Unilateral hematoma	1 (1.6)

## Discussion

In general, normal breasts, an important aesthetic organ for females, are characterized as hemispherical, soft to touch, and have the same volume on both sides (7). However, congenital dysplasia, breastfeeding, and poor exercise posture can lead to breast atrophy, sagging, and even obvious asymmetrical size on both sides, seriously affecting the appearance of female breasts (8). Breast augmentation with breast prosthesis is the most widely used breast plastic surgery to promote breast enlargement and restore the original symmetry. The main parameters of design before breast augmentation include available skin and soft tissue coverage area, the volume and size of implanted prosthesis, the position of the inframammary fold, and the site of incision (9). At the same time, the occupation, height, and body shape of patients should be fully considered.

Dual-plane, subglandular, subpectoral, and subfascial breasts were performed as common implant pockets. The high incidence of capsular contracture limits the application of the subglandular augmentation (10, 11). Strasser et al. (12) found that the subglandular augmentation compared with subpectoral augmentation showed different degrees of capsular contracture and implant appearance depending on the type of implant and the volume of breast tissue. After subpectoral augmentation, the pectoralis major can cover 60 to 75% of the upper part of the prosthesis, and the lower lateral part of the prosthesis only is directly exposed to the rear of the breast (13). Compared with the subglandular augmentation, the subpectoral augmentation increases the coverage of the pectoralis major so that it can significantly reduce the exposure rate of prosthesis edge, and the incidence of capsular contracture as well (10, 14). However, subpectoral mammoplasty also has certain defects, such as the postoperative breast feel is not soft enough and the prosthesis is prone to displacement due to the surface tension and contraction of the pectoralis major. For patients with markedly mastoptosis, bimastism may occur after surgery (15). Meanwhile, subpectoral mammoplasty requires the stripping of the pectoralis major, which is traumatic and performed under blind vision with the bleeding may not be completely stopped leading to potential hematoma or massive hemorrhage. Therefore, the drainage tube needs to be placed for a long time and the recovery time is prolonged (16). Subfascial mammoplasty is difficult and cannot significantly reduce complications. Brown et al. (17) followed up 83 submammary breast augmentation patients and 200 subfascial breast augmentation patients for up to 51 months and found no significant difference in patient satisfaction and complications between the two groups, so further exploration is needed.

Dual-plane mammoplasty, first proposed by Tebbetts et al. (18), refers to severing the attachment point of the pectoralis major around the inframammary fold, retaining the starting point of pectoralis major at the sternum. After the operation, the upper part of the prosthesis is covered by the pectoralis major, while the lower part is directly behind the breast. Biplane augmentation mammoplasty allows the prosthesis placed behind the breast and pectoralis major at the same time with many advantages of two planes, as follows: (1) the ribs, sternum, anterior axillary line, internal thoracic artery intercostal perforator, and intercostal nerve perforator can serve as anatomical landmarks for the prosthesis placement cavity during the stripping process, which is helpful for accurate stripping of the implanted cavity, protection of nerve function and adequate hemostasis to obtain good breast augmentation shapes and reduce the incidence of postoperative complications. (2) Dual-plane augmentation mammaplasty needs to strip the attachment point of pectoralis major around the inframammary fold, which decreases the compression effect of the pectoralis major, thereby greatly moderating postoperative pain, and reducing the incidence of postoperative prosthesis displacement and bimastism (19). (3) The severed pectoralis major drives the mammary glands to retract upwards, so that there is more tissue coverage on the prosthesis to the tissue plumpness of the upper breast is increased, which significantly reduces the visibility of the prosthesis edge. Similarly, the lower part of the prosthesis is directly behind the breast and was covered by the new inframammary fold to obtain a natural inframammary fold and avoid direct contact with the contour of the prosthesis.

Commonly used incisions for breast implants are subareolar incision, transaxillary incision, and inframammary fold incision. Although the surgical path of the inframammary fold incision is short and the cavity can be dissected easily and hemostasis can be performed under direct vision, the postoperative scar is obvious so that most patients are unwilling to choose, especially for Asian patients. The anterior cutaneous branch of the fourth intercostal nerve is the most stable nerve innervating the nipple and areola sensation, and avoiding the skin incision at the inner edge of the areola will protect the nerve innervating the nipple and areola to the greatest extent (20). Thus, a subareolar incision provides a good surgical field of vision, but the incidence of paresthesia in the nipple and areola area is higher. Moreover, there are many bacteria around the mammary opening, leading to the risk of infection and capsular contracture. For patients with light areola, scars after subareolar incision are obvious, and some patients are reluctant to accept it (13). Compared with the above incisions, the position of the transaxillary incision is hidden with the postoperative scars are not easy to see, but the operation path is far away and the field is not clear so that most of the operations are performed under blind vision, which makes it difficult for the surgeon to predict the boundaries of pectoralis major dissection or produce obvious inframammary fold, and complications such as hematoma and trauma are prone to cause (6, 21). Especially, the traditional transaxillary breast augmentation under blind vision cannot complete the establishment of a biplane implant cavity. Interestingly, the endoscope used in transaxillary breast augmentation could greatly avoid above defects. Endoscope technology transforms traditional breast augmentation from blind vision to direct vision, which makes the anatomical structure of mammoplasty clearer and hemostasis more thorough. For the former, the position of the inframammary fold and the pectoralis major, the perforator of the internal thoracic artery, and the perforator of the intercostal nerve can be more accurately observed through the endoscope, so as to better control the transaction plane and obtain a good appearance of breast augmentation and reduce postoperative complications. For the latter, the bleeding site can be seen under the endoscope to hemostasis accurately. Meanwhile, the surgeon can accurately peel off the cavity, which greatly reduces the damage to the surrounding tissues by the operation, and reduces the incidence of postoperative hematoma and long-term capsular contracture, the postoperative pain, and hospitalization time of the patients.

**Figure 7:**
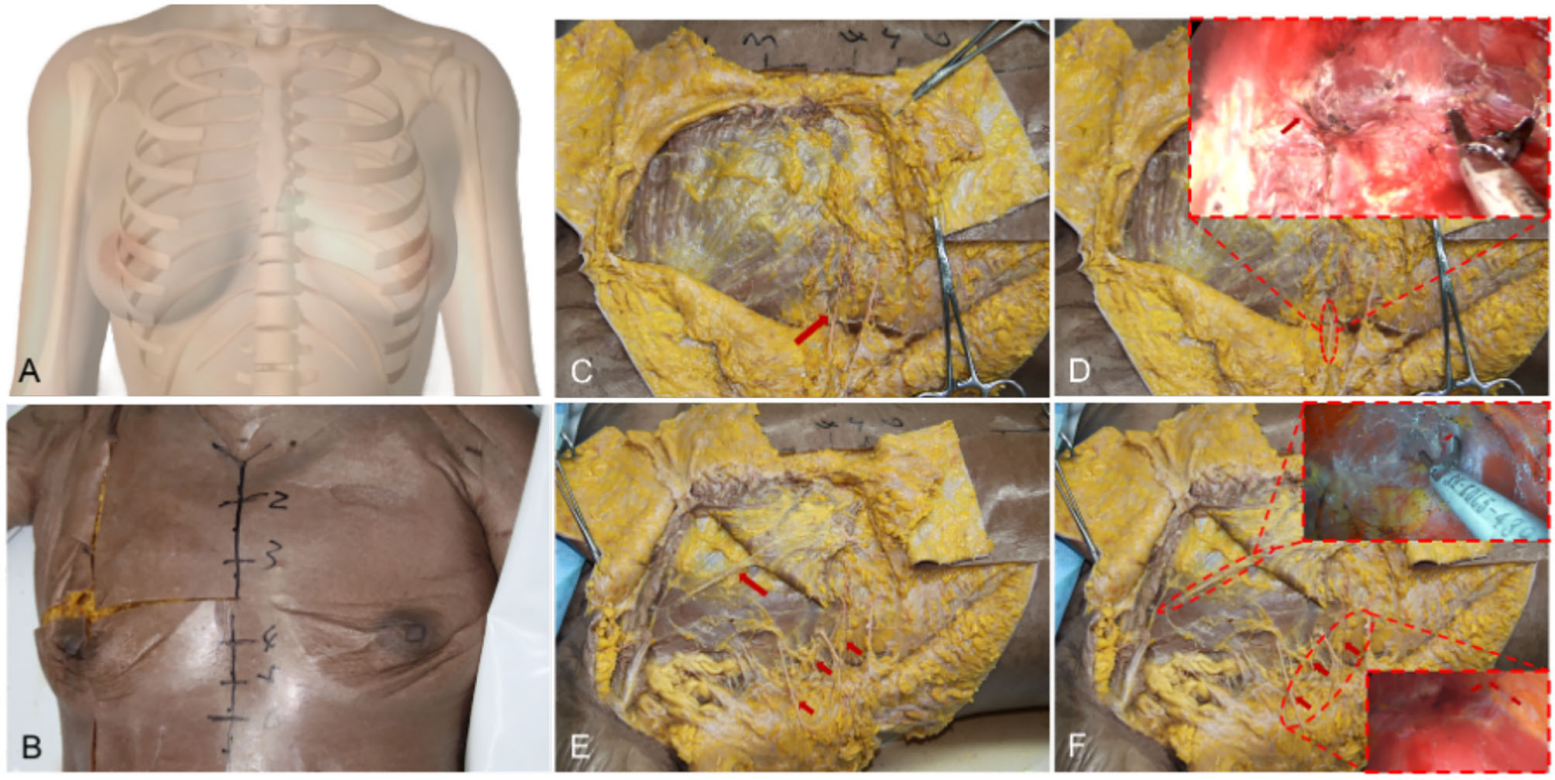
General anatomy of the breast and the vessels anatomy of the lateral and upper pocket. **(A)** A schematic diagram shows the adult female breasts. **(B)** Breast general anatomy showed the approximate location of the adult female breasts. **(C)** Arrow indicates the anterior cutaneous branch of the fourth intercostal nerve. **(D)** The anterior cutaneous branch of the fourth intercostal nerve needs to be carefully protected combined with an endoscope. **(E)** Arrows indicate a deep blood vessel from the pectoralis minor to the pectoralis major in the upper pocket and a set of intercostal nerves and their accompanying blood in the upper pocket. **(F)** Vascular anatomy of lateral and upper pocket combined with an endoscope.

**Figure 8:**
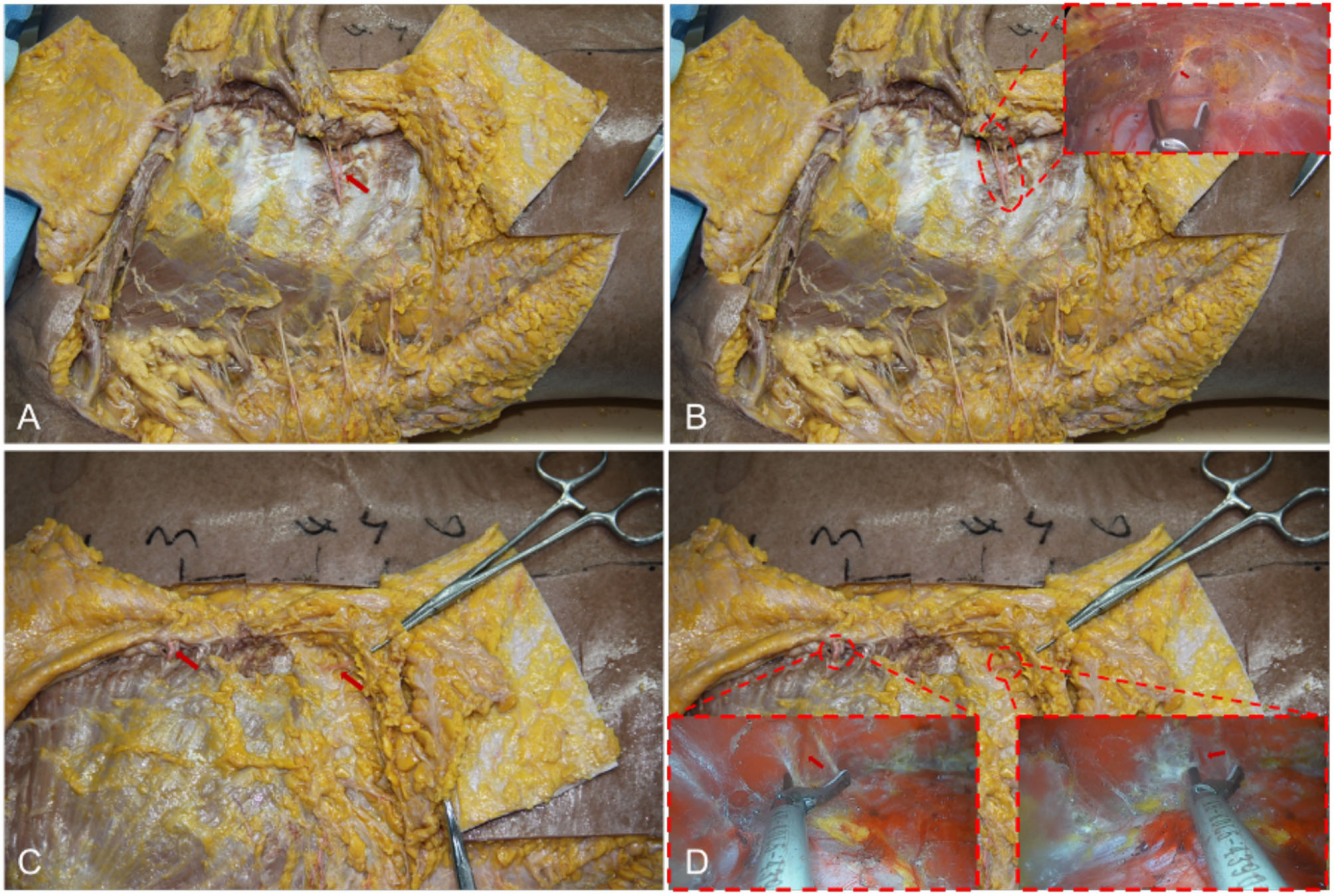
The vessels' anatomy of the lower and lateral pocket. **(A)** Arrow indicates a vascular bundle from intercostal to pectoralis major muscle in the fourth intercostal space. **(B)** Vascular anatomy of lower pocket combined with an endoscope. **(C)** Arrows indicate a group of vascular and nerve bundles in the 2–3 intercostal and 4–5 intercostal spaces on the lateral edge of the sternum. **(D)** Vascular anatomy of lateral pocket combined with an endoscope.

However, there is no uniform instruction for transaxillary endoscopic biplane breast augmentation currently. If the surgeons do not fully grasp the breast anatomical structure, it will greatly increase the difficulty of the operation and postoperative complications. In this study, we propose the breast anatomical structure to guide transaxillary endoscopic biplane breast augmentation to achieve good results and is suitable for all prophylactic and therapeutic prosthetic breast augmentation patients, including patients with cancer and skin/nipple-sparing mastectomy. Adult female breasts are mostly located in the second or third rib to the sixth rib, inner reaches the sternum, and outer to the anterior axillary line (Figures 7A,B). Previous studies have reported that the blood supply of the breast mainly comes from the internal thoracic artery, the axillary artery branch, and the deep intercostal artery (22–24). Firstly, we can observe that there is a deep blood vessel from the pectoralis minor to the pectoralis major near the second rib of the midclavicular line (Figure 7E). Therefore, the second rib must not be exceeded when the upper boundary of the cavity is created. Secondly, we can observe a set of intercostal nerves and their accompanying blood vessels from the deep surface of the pectoralis major to the breast at the level of the anterior axillary line on the lateral border of the pectoralis major (Figures 7C,E). And the anterior cutaneous branch of the fourth intercostal nerve has the function of innervating the sensation of the nipple and areola, which needs to be carefully protected (Figure 7D) (24–28). According to the above anatomic location, when intercostal nerves and vascular perforators are observed, which indicates that the cavity has been separated to the lateral boundary, and the vascular perforators must be fully disconnected (Figure 7F). At the same time, the intercostal nerves need to be protected, especially to avoid damaging the anterior cutaneous branch of the fourth intercostal nerve, in order to prevent postoperative sensory disturbance of the nipple areola. Thus, none of the patients in this study had these complications. Thirdly, we observed a vascular bundle from intercostal to pectoralis major muscle in the fourth intercostal space of the midclavicular line (Figure 8A). During the preparation of the prosthesis implantation cavity, the vascular bundle can be observed and needs to be performed disconnection and hemostasis to reduce the possibility of postoperative hematoma (Figure 8B and Supplementary Video 4). Fourthly, a group of vascular and nerve bundles in the 2–3 intercostal and 4–5 intercostal spaces on the lateral edge of the sternum (Figure 8C), helps to locate the medial boundary of the implanted cavity. Similarly, the intercostal vascular bundles need to be disconnected (Figure 8D and Supplementary Videos 5, 6). In general, under the guidance of basic anatomy, endoscopic biplane breast augmentation can effectively minimize the risks of breast flap necrosis and nipple and areola sensory disturbances and featured immediate reconstruction.

**Figure 9:**
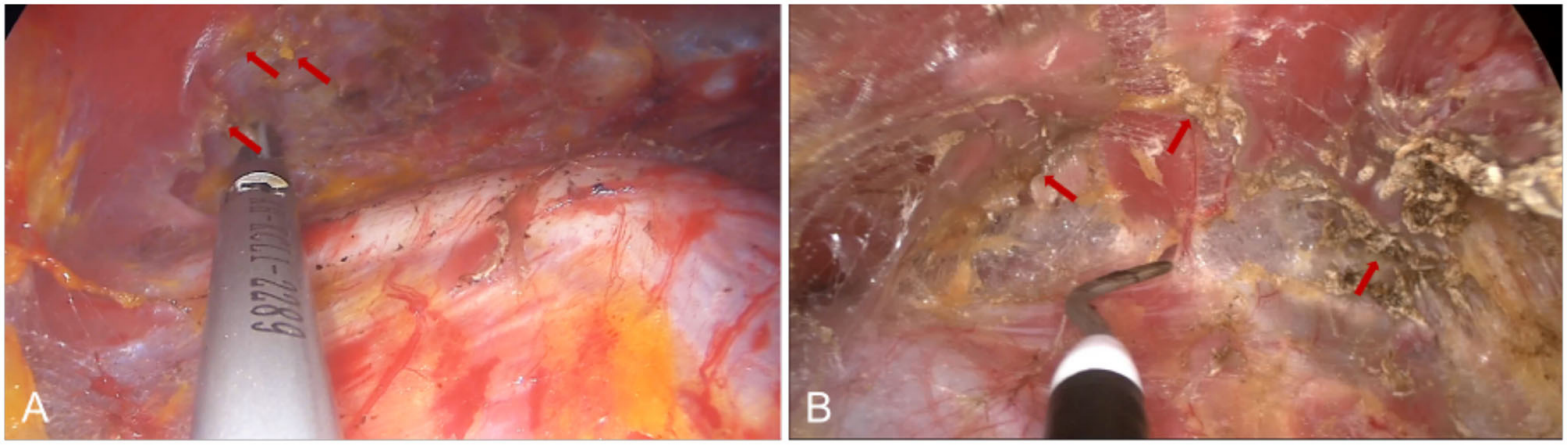
Comparison of tissue cutting effects between ultrasonic scalpel and electric hook. **(A)** Arrows indicate small tissue damage after ultrasonic knife cutting tissue. **(B)** Destructive tissue damage after electric hook cutting.

Traditional transaxillary endoscopic biplane breast augmentation almost uses electrical hooks and bipolar electrocoagulation forceps for tissue separation and hemostasis. However, the scope of thermal damage to the surrounding tissue by electric hook and bipolar coagulation forceps is large and obvious (Figure 9B). In this study, the ultrasound knife was performed in the transaxillary endoscopic biplane breast augmentation. The ultrasonic knife converts the electrical energy to the ultrasonic engine through the transducer to make the metal probe mechanically oscillate at an ultrasonic frequency of 55.5 kHz and drive high-frequency oscillations in the tissue cells, so that the hydrogen bond of the protein in the cells breaks, the tissue collapses, and the denatured protein forms a gel to seal the blood vessels to achieve the hemostatic effect (29). Compared with the electric hook and bipolar electrocoagulation forceps, the ultrasonic knife has more accurate temperature control (Figure 9A), which can simultaneously cut and coagulate vessels, large tissue pedicle and vascular bundle in the case of minimal tissue thermal injury to significantly reduce the postoperative burning sensation of patients (30). We recommend that in the establishment of a biplane cavity combined with an ultrasonic knife. On the one hand, the ultrasonic scalpel has little damage to tissues and separates the range of the biplane cavity and the attachment point of the pectoralis major around the inframammary fold more accurately, thus speeding up the postoperative recovery and having a good postoperative experience of patients. On the other hand, if intraoperative hemostasis is not complete, postoperative bleeding and oozing of blood will have a serious impact on the surgical effect. For instance, the probability of complications such as postoperative hematoma, infection, and capsular contracture will greatly increase, and the drainage tube placement time and hospital stay will be prolonged, while combined with an ultrasonic scalpel to get a more thorough intraoperative hemostasis effect. This may be one of the reasons why the patients in this study were able to remove the drainage tube and be discharged from the hospital on the next day after surgery, achieving no hematoma complications and high satisfaction. In addition, the use of endoscopy and ultrasonic knives will inevitably increase the cost of surgery compared to traditional breast augmentation, but this cost only accounts for about 14% of the total cost. Moreover, endoscopy combined with an ultrasonic knife, does not significantly increase the operative time, can adequately stop bleeding and separate cavity with good surgical vision, thus effectively reducing post-operative complications and improving post-operative comfort, which patients are willing to accept.

Capsular contracture is a common complication of breast augmentation. Previous studies (31, 32) have reported that capsular contracture is related to factors such as hematoma and infection, thus effective control of hematoma is beneficial to reduce the incidence of long-term capsular contracture. In this study, the combined application of endoscope and ultrasonic knife establishes a biplane implantation cavity, which can fully stop bleeding under direct vision, and routine postoperative placement of drainage can reduce the incidence of hematomas effectively. At the same time, the application of conveyor belts also greatly reduces the possibility of infection. Implant malposition is an important cause of postoperative asymmetry of breasts, which will seriously affect the results and patient satisfaction after breast augmentation. Implant malposition is mainly due to compression of the pectoralis major muscle, insufficient separation around the insertion of the pectoralis major at the lower border of the cavity, and excessive dissection on the upper and medial sides of the cavity (16, 33). In this study, biplane breast augmentation strips the attachment points of the pectoralis major around the inframammary fold, reducing the squeezing effect of the pectoralis major. At the same time, we determine the appropriate range of dissection by the anatomical structure of the breast to avoid the occurrence of this complication. Infection is an important cause of unplanned secondary surgery or even removal of the prosthesis, with an incidence of about 1.1% (34, 35). Eid et al. (36) found that the use of non-contact techniques for implantation of breast prostheses reduced the incidence of infection to zero. This is consistent with the findings of this study.

However, this study still has certain limits. Previous studies (37–39) have found that the incidence of capsular contracture after transaxillary breast augmentation is 1 to 7%. The experience of the surgeon, the number of patients, and the follow-up time are related to the rate of capsular contracture (38, 39). Only one complication of early capsular contracture was observed in this study, we think it is caused by the limitation of follow-up time and the number of study samples, so we plan to accumulate more cases and have a longer follow-up time to record. Meanwhile, we did not set up traditional breast augmentation as a control group. Follow-up studies can collect relevant data of traditional breast augmentation patients and conduct follow-up observations to make the research results more scientific.

## Conclusion

In summary, transaxillary endoscopic biplane breast augmentation can obtain a good surgical field, and the application of an ultrasonic knife combined with breast anatomy can effectively close the blood vessels and avoid damage to important nerves to reduce the complications of postoperative hematoma and nipple-areola sensory disturbances on the basis of obtaining a good breast shape, which is worthy of clinical application.

## Data Availability Statement

The raw data supporting the conclusions of this article will be made available by the authors without undue reservation.

## Ethics Statement

Written informed consent was obtained from the individual(s) for the publication of any potentially identifiable images or data included in this article.

## Author Contributions

HJ and HW: designed the study. JX, ZH, QH, and YG: performed and drafted the experiment. LL and MS: revised the manuscript. All authors participated in the operation and approved the final version of the manuscript.

## Funding

This work was supported by the East Hospital affiliated with Tongji University introduced a talent research startup fund (Grant Number DFRC2019008), and the featured clinical discipline project of Shanghai Pudong (Grant Number WYts2021-07).

## Conflict of Interest

The authors declare that the research was conducted in the absence of any commercial or financial relationships that could be construed as a potential conflict of interest.

## Publisher's Note

All claims expressed in this article are solely those of the authors and do not necessarily represent those of their affiliated organizations, or those of the publisher, the editors and the reviewers. Any product that may be evaluated in this article, or claim that may be made by its manufacturer, is not guaranteed or endorsed by the publisher.
